# Human Mesenchymal Stem Cell Expression Program upon Extended *Ex-Vivo* Cultivation, as Revealed by 2-DE-Based Quantitative Proteomics

**DOI:** 10.1371/journal.pone.0043523

**Published:** 2012-08-20

**Authors:** Andreia Madeira, Cláudia L. da Silva, Francisco dos Santos, Emilio Camafeita, Joaquim M. S. Cabral, Isabel Sá-Correia

**Affiliations:** 1 Institute for Biotechnology and Bioengineering (IBB), Centre for Biological and Chemical Engineering, Instituto Superior Técnico, Lisboa, Portugal; 2 Department of Bioengineering, Instituto Superior Técnico, Technical University of Lisbon, Lisboa, Portugal; 3 Unidad de Proteómica, Centro Nacional de Investigaciones Cardiovasculares, CNIC, Melchor Fernández Almagro 3, Madrid, Spain; Universidade do Porto, Portugal

## Abstract

Human mesenchymal stem cells (MSC) have been on the focus of intense clinical-oriented research due to their multilineage differentiation potential and immunomodulatory properties. However, to reach the clinically meaningful cell numbers for cellular therapy and tissue engineering applications, MSC *ex-vivo* expansion is mandatory but sequential cell passaging results in loss of proliferative, clonogenic and differentiation potential. To get clues into the molecular mechanisms underlying cellular senescence resulting from extended *ex-vivo* cultivation of bone marrow (BM) MSC, we explored a two-dimensional gel electrophoresis (2-DE) based quantitative proteomics to compare the expression programs of Passage 3 cells (P3), commonly used in clinical studies with expanded MSC, and Passage 7 (P7) cells, which already demonstrated significant signs of culture-induced senescence. Proteins of the functional categories “Structural components and cellular cytoskeleton” and “Folding and stress response proteins” are less abundant in P7 cells, compared to P3, while proteins involved in “Energy metabolism”, “Cell cycle regulation and aging” and “Apoptosis” are more abundant. The large number of multiple size and charge isoforms with an altered content that were identified in this study in P7 *versus* P3, namely the cytoskeleton components β-actin (7 forms) and vimentin (24 forms), also emphasizes the importance of post-transcriptional modification upon long-term cultivation. The differential protein expression registered suggests that cellular senescence occurring during *ex-vivo* expansion of BM MSC is associated with the impairment of cytoskeleton remodeling and/or organization and the repair of damaged proteins resulting from cell exposure to culture stress. The genome-wide expression approach used in this study has proven useful for getting mechanistic insights into the observed decrease on the proliferative and clonogenic potential of P7 *versus* P3 cells and paves the way to set up a proteome profiling strategy for quality control to assure safe and clinically effective expanded MSC.

## Introduction

Mesenchymal stem cells (MSC) are multipotent stem cells with self-renewal capacity and the ability to differentiate into osteoblasts, chondrocytes, and adipocytes, among other mesenchymal cell lineages. In recent years, the intense research on the multilineage differentiation potential and immunomodulatory properties of human MSC have indicated that these cells can be used to treat a range of clinical conditions, including immunological disorders as well as degenerative diseases [1]. Consequently, the number of clinical studies with MSC has been steadily increasing for a wide variety of conditions: graft-*versus*-host disease (GVHD) [2], bone and cartilage defects [3], myocardial infarction [4] and autoimmune diseases [5], among others. The high cell doses required for MSC clinical applications (up to several million cells per kg of the patient [2]) demands a reliable, reproducible and efficient expansion protocol, capable of generating a large number of cells. The currently used clinical-scale MSC expansion protocol is based on traditional cell culture techniques where MSC are cultured into plastic tissue culture flasks, which are limited in terms of cell productivity and demand extensive handling for cell culture processing (*e.g.* medium renewal). At least 2 to 3 cell passages are commonly required to achieve clinically relevant cell numbers in an acceptable period of time [6] and a rigorous set up of cell characterization assays to assure a safe and clinically effective MSC product is critical. Human MSC are commonly defined by: i) their plastic adherence in culture, (ii) specific surface antigen expression, and (iii) multilineage *in vitro* differentiation potential [7]. As during aging *in vivo*, sequential *ex-vivo* cell passaging might be associated with replicative stress, chromosomal abnormalities, or other stochastic cellular defects, resulting in the progressive loss of the proliferative, clonogenic and differentiation potential of the expanded cells [8], which ultimately can jeopardize MSC clinical safety and efficacy. The use of senescent cells in clinics should not be underestimated since cells lose part of their differentiation potential and their secretory profile is also altered [9]. MSC senescence during culture was found to induce cell growth arrest, with telomere shortening [10] and a continuous decrease in adipogenic differentiation potential was reported for bone marrow (BM) MSC along increasing passages, whereas the propensity for differentiation into the osteogenic lineage increased [11]. Overall, MSC senescence is a complex, finely organized process at genomic, transcriptomic, epigenetic and proteomic levels [9]. Standardized biomarkers based on specific molecular targets to attest the functionality (*i.e.* differentiative potential and immunomodulation), as well as safety of MSC upon long-term *ex-vivo* cultivation are largely needed. Genes or molecules involved in senescence pathways, known to be up-regulated by senescence signals [12], are of potential use for these biomarkers. In recent years, quantitative proteomics has emerged as a genome-wide expression approach for the proteome profiling of MSC cells and the identification of protein networks involved in proliferation and differentiation under different experimental conditions [12], [13], [14]. Quantitative proteomic analysis based on two-dimensional gel electrophoresis (2-DE) allows the quantitative analysis of proteomes also taking into account the extensive molecular variety of protein forms resulting, in eukaryotes, from alternative splicing, mRNA editing or co- and post-translational modifications, thus providing comprehensive data at a molecular system biology level [15].

Considering the growing clinical applications of expanded BM MSC, we have been focused on the optimization of *ex-vivo* culture conditions for human MSC expansion, namely by using a low oxygen environment (2%) [16] or a microcarrier-based dynamic culture system [17] operating under xenogeneic-free conditions [18]. However, there is an increasing knowledge that long-term *ex-vivo* cultivation has to be taken into account in order to avoid alterations in the efficacy and safety of the cellular product. Indeed, most of the reported clinical studies with expanded MSC used cells expanded up to a maximum of 3 or 4 passages [6]. This was also the case of the MSC infusions used in the first clinical studies with *ex-vivo* expanded cells (2–3 passages were used) in Portugal to treat GVHD and as adjuvants of hematopoietic cell transplantation ([19] and dos Santos, PhD Thesis, 2011). However, as previously described, a comprehensive control panel to attest MSC product quality is still to be defined.

In the present study we explored a 2-DE based quantitative proteomic approach to address the impact of extended *ex-vivo* cultivation involving consecutive passaging on human BM MSC. To this end, cells from a BM donor were *ex-vivo* expanded on tissue culture flasks and at Passage 3 (P3) (an early passage commonly used in clinical settings) and at Passage 7 (P7) the proteomes of cells were separated by 2-DE, protein spots were detected by fluorescent gel staining, the gels were scanned and the obtained images were analyzed using a dedicated software to get a snapshot of the alterations of the MSC proteome, as described before [20], [21], [22]. Passage 7 was selected since these cells already presented signs of culture-induced senescence. However, P7 represents an “earlier” passage compared to others referred in literature as “late passages” namely P8 [23] or P12 [11], [24], for which the occurrence of replicative senescence was demonstrated. The exploitation of this global expression approach and design have proven useful for unveiling the molecular mechanisms underlying the observed decrease on proliferative and clonogenic potential upon consecutive passages and pave the way to establish a proteomic analysis platform as a quality control for MSC products towards the development of safer and more effective cell therapies.

## Results

### Quantitative Assessment of the Loss of Proliferative Potential and Clonogenic Ability of BM MSC Population along Consecutive Passages

In order to quantitatively assess the effects of consecutive cell passaging on the proliferative and clonogenic potential of human BM MSC, samples of cells independently isolated from 4 different healthy donors (Donors 1–4) were cultured for 9 consecutive passages (P1 to P9) using MSC-qualified DMEM supplemented with 10% FBS. At each passage, BM MSC were seeded at 3×10^3^ cells/cm^2^ and harvested and re-plated at 80% confluence, and the specific growth rate, population doublings, clonogenic potential and differentiation potential were evaluated.

**Figure 1:**
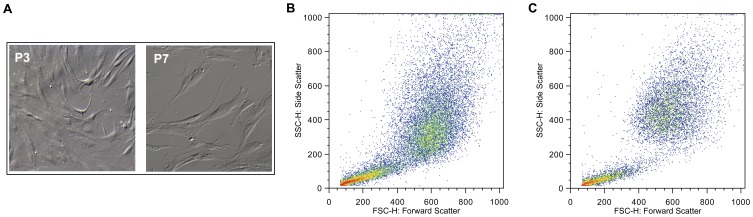
Morphological changes upon senescence. Representative morphology of passage 3 (P3) and passage 7 (P7) BM MSC with a 100X magnification is presented (A). The increase in cell size and granularity is reflected by the increasing of the forward- and side-scatter values assessed by flow cytometry for the BM MSC cultured cells at P3 (B) and P7 (C).

**Figure 2:**
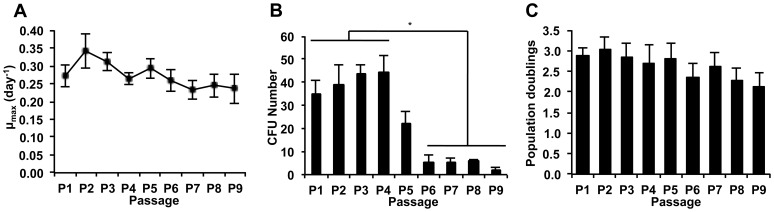
Quantitative assessment of MSC proliferative potential, clonogenic ability and population doublings. (A) Proliferative analysis measured during the *ex-vivo* expansion of the BM MSC, determined by the Trypan Blue exclusion method; (B) Colony forming units-fibroblast (CFU-F) assays performed at day 7 for each passage; (C) Population doublings calculated during time in culture.

**Figure 3:**
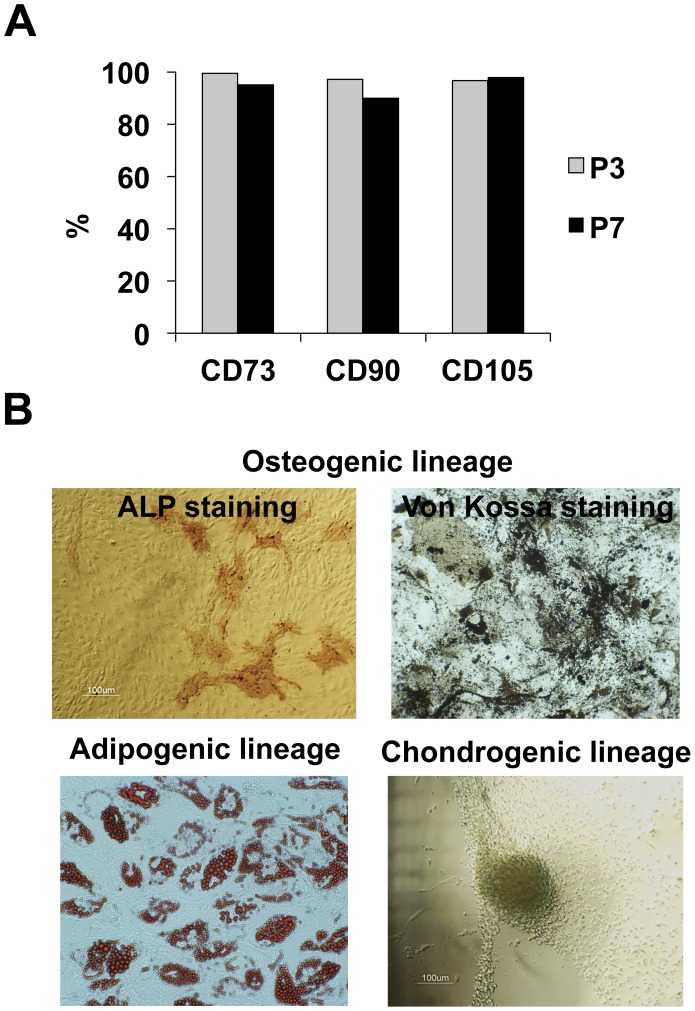
MSC immunophenotyping and multilineage differentiation potential. (A) Analysis of surface antigen marker by flow cytometry for cells obtained from Donor 1. Cells were stained with CD73, CD90 and CD105 antibodies. The IgG isotype was used as control; (B) Ability to differentiate into several lineages: Osteogenic differentiation was indicated by the ALP and Von Kossa staining (upper panel). Adipogenic differentiation (left lower panel) is visually marked by accumulation of neutral lipid vacuoles in culture (Oil Red-O staining). Chondrogenic differentiation (right lower panel) is visually marked by Alcian Blue staining.

Morphological changes of cells during the consecutive passaging expansion were observed. In particular, the comparison of images of cell culture in passages 3 and 7 (Fig. 1A) showed an enlargement of BM MSC in culture, as well as a visible increase of cell granularity, for cells in a late passage. Concomitantly, the analysis of flow cytometry data (Fig. 1B and 1C) reveals higher values of forward scatter and side-scatter for late passage cells, indicating an increased size and higher level of morphologic complexity, respectively, similarly to a previous study [11]. In terms of cell proliferation, results show that the specific growth rate of BM MSC achieved maximum values on passages 2 and 3 (0.34±0.05 day^−1^ and 0.31±0.03 day^−1^), decreasing in subsequent passages (Fig. 2A). The clonogenic potential of BM MSC was also affected by consecutive passaging: while in the first 4 passages the CFU number obtained increased slightly, with an average of 40±4 CFU, a significant decrease was observed upon passage 5, with an average of 5±2 CFU for passages six to nine (Fig. 2B) (*p*≤0.05). Concomitantly, a decreasing trend was observed for population doublings as passage number increased (Fig. 2C). Overall, these results point towards the combined loss of proliferative and clonogenic potential of BM MSC along multiple and consecutive cell passaging, especially evidenced upon passages 4–5.Interestingly, within the cell passages studied (P1 to P9), BM MSC maintained their characteristic immunophenotype (over 90% positive for CD73, CD90 and CD105) [7] (Fig. 3A) and multilineage differentiation potential (MSC were able to differentiate into osteogenic, adipogenic and chondrogenic lineages) (Fig. 3B). However, the level of expression (mean intensity) of surface antigens, namely CD73 and CD105, was higher in lower passages (mean of fluorescence intensity for P3 cells: 225 for CD73 and 75.1 for CD105) compared to later passages (mean of fluorescence intensity for P7 cells: 116 for CD73 and 54.3 for CD105), suggesting an attenuation in the expression level of these surface receptors along long-term cultivation.

### Differential Protein Expression Profiles of P3 and P7 Cells

**Figure 4:**
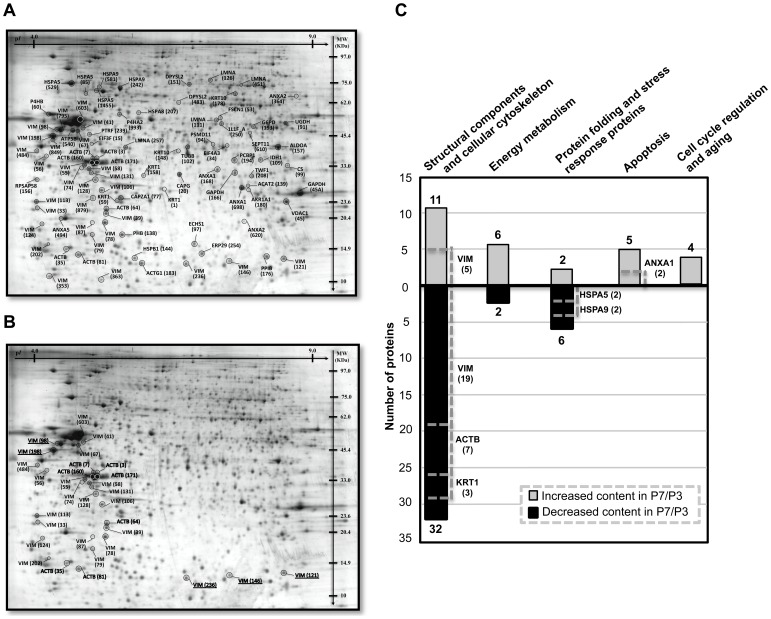
Differential protein expression of P3 and P7 cells. (A) 2-DE reference map of the human BM MSC obtained from Donor 1; (B) protein map showing the position of the 7 ACTB and of the 24 VIM isoforms showing a higher (underlined) or a lower content in P7 compared to P3; (C) functional categories with the highest number of proteins whose content is altered in P7 compared to P3. The number of protein isoforms is indicated.

The quantitative proteomic analysis, based on 2-DE, was performed with cells retrieved from passage 3 (P3) and passage 7 (P7). These cells were isolated from one of the 4 single BM healthy donors (Donor 1) and cultured in 3 independent *ex-vivo* cultures, along consecutive passages, since P2 until P7. A p*I* range spanning from pH 4 to close to pH 9 was used in 2-DE and a reference map was prepared for the proteins present in the mixture of P3 and P7 samples. A representative gel is shown in Fig. 4A. On average, a total of 2500 spots were separated in analytical gels loaded with 100 µg of total protein extract of each sample (P3 or P7), of which 199 spots were selected for identification, most of them due to their altered content in P3 *versus* P7 samples. The ANOVA values for each protein spot were calculated as the average data from three replicates of each sample and data was filtered to retain spots with ANOVA *p*<0.05. The spots were excised manually from silver-stained preparative gels and 89 protein spots were identified by mass spectrometry (MS), corresponding to a 45% of succeeded identification. The protein amount of part of the protein spots that failed identification was below the detection level. Of the 79 identified protein spots whose relative abundance was considered to vary in P7 compared to P3 (fold change ≥1.3; *p*≤0.05; ANOVA), 30 are unique protein forms while the remaining correspond to multiple charge and size isoforms of 9 different proteins, presumably due to alternative splicing and/or post-translational modifications (PTM): β-actin (ACTB), keratin, type II cytoskeletal 1 (KRT1), keratin, type I cytoskeletal 10 (KRT10), vimentin (VIM), 78 kDa glucose-regulated protein (HSPA5), heat shock 70 kDa protein 9 (HSPA9), annexin A1 (ANXA1), lamin A/C (LMNA) and dihydropyrimidinase-like 2 (DPYSL2) (Table 1). In particular, proteins ACTB and VIM exhibit the highest number of different isoforms, 7 and 24, respectively (Table 1, Fig. 4B).

**Table 1:** List of the identified proteins with differential expression in P7 by comparison with P3.

Spot (nr.)	Protein function	Accession Number	MW (KDa)^a^/p*I* b	Mascot score	No. of matched peptides	Sequence coverage (%)		Fold change (P7/P3)	ANOVA (p)^c^
**Structural components and cellular cytoskeleton**									
3	actin, cytoplasmic 1; beta-actin (**ACTB**)	P60709	35.6/5.29	243	5	18	−5.4		0.035
7			35.6/5.29	244	6	21	−3.8		0.027
35			15.1/5.29	144	4	13	−2.5		0.049
64			25.0/5.29	158	3	12	−2.1		0.004
81			14.3/5.29	116	4	13	−2.0		0.030
160			35.0/5.29	356	15	42	−1.6		0.041
171			35.0/5.29	97	5	18	−1.5		0.040
1	keratin, type II cytoskeletal 1 (**KRT1**)	P04264	27.2/8.15	154	12	24	−9.5		0.005
59			24.3/8.15	96	8	17	−2.1		0.013
75			97.2/8.15	58	5	9	−2.0		0.050
158			30.7/8.15	91	8	15	1.6		0.048
148	keratin, type I cytoskeletal 10 (**KRT10**)	P13645	38.7/5.13	85	7	10	−1.6		0.038
178			68.3/5.13	95	8	18	1.5		<0.001
33	vimentin (**VIM**)	P08670	22.8/5.06	128	7	23	−2.5		0.010
39			21.2/5.06	277	17	29	−2.4		0.011
41			48.9/5.06	249	16	44	−2.4		0.020
55			34.0/5.06	497	21	41	−2.1		0.015
56			37.7/5.06	258	13	34	−2.1		0.009
58			33.1/5.06	403	24	49	−2.1		0.013
67			47.1/5.06	294	16	39	−2.1		0.022
74			33.1/5.06	227	15	37	−2.0		0.029
78			19.3/5.06	406	22	34	−2.0		0.002
79			17.2/5.06	204	9	21	−2.0		0.049
87			19.3/5.06	82	3	7	−2.0		0.009
106			26.9/5.06	173	13	29	−1.8		0.044
113			23.7/5.06	248	12	27	−1.8		0.010
124			19.3/5.06	160	10	30	−1.7		0.026
128			29.6/5.06	103	6	16	−1.7		0.018
131			31.9/5.06	400	18	39	−1.7		0.007
202			16.0/5.06	164	9	27	−1.4		0.010
484			39.1/5.06	318	20	45	−1.8		0.050
603			53.6/5.06	713	33	63	−1.6		0.052
98			48.9/5.06	157	15	46	1.9		0.014
121			14.1/5.06	192	11	29	1.7		0.012
146			14.1/5.06	157	9	24	1.6		0.025
198			44.5/5.06	883	29	59	1.4		0.008
236			13.1/5.06	101	6	16	1.3		<0.001
20	macrophage-capping protein (**CAPG**)	P40121	31.0/5.88	158	6	23	2.9		0.004
53	fascin homolog 1, actin bundling protein (*Strongylocentrotus purpuratus*) (**FSCN1**)	Q16658	54.1/6.84	121	7	17	2.2		0.011
77	capping protein (actin filament) muscle Z-line, alpha 1 (**CAPZA1**)	P52907	24.8/5.45	115	6	24	−2.0		0.017
102	tubulin beta chain (**TUBB**)	P07437	40.6/4.78	161	9	19	1.8		0.001
183	actin, gamma 1 (**ACTG1**)	P63261	13.1/5.65	136	3	16	−1.5		0.010
208	twinfilin, actin binding protein (**TWF1**)	Q12792	30.7/6.37	149	4	20	1.4		0.031
**Folding of proteins and stress response proteins**									
60	protein disulfide-isomerase (P55) (**P4HB**)	P07237	56.2/4.76	89	6	11		−2.1	0.040
85	78 kDa glucose-regulated protein (**HSPA5**)	P11021	68.3/5.07	266	7	14		−2.0	0.010
529			74.9/5.07	243	10	18		−1.5	0.198
242	heat shock 70 kDa protein 9 (mortalin) (**HSPA9**)	P38646	72.9/5.87	166	7	13		−1.3	0.009
581			72.9/5.87	111	7	15		−1.6	0.070
144	heat shock protein beta-1 (HSP 27) (**HSPB1**)	P04792	18.8/5.98	113	3	20		−1.6	0.002
176	peptidyl-prolyl cis-trans isomerase B (cyclophilin B) (**PPIB**)	P23284	14.1/9.42	93	6	30		1.5	0.017
207	heat shock cognate 71 kDa protein (**HSPA8**)	P11142	56.7/5.37	227	9	18		1.4	0.006
**Apoptosis**									
168	annexin A1 (**ANXA1**)	P04083	32.5/6.57	351	14	49		1.5	0.033
698			27.2/6.57	307	17	50		1.5	0.053
364	annexin A2 (**ANXA2**)	P07355	66.4/7.57	143	8	25		2.4	0.068
494	annexin A5 (**ANXA5**)	P08758	20.2/4.94	121	6	20		1.8	0.060
45	voltage-dependent anion-selective channel protein 1 (**VDAC1**)	P21796	23.9/8.62	144	3	12		2.2	0.025
**Vesicle trafficking**									
610	septin 11 (**SEPT11**)	Q9NVA2	46.6/6.36	105	6	15		1.6	0.059
**Cell cycle regulation and aging**									
15	eukaryotic translation initiation factor 3, subunit F (**eIF3f**)	O00303	41.7/5.24	99	5	19		3.1	0.039
34	eukaryotic translation initiation factor 4A3 (**eIF4A3**)	P38919	40.9/6.30	154	12	24		2.5	0.003
94	proteasome (prosome, macropain) 26S subunit, non-ATPase, 11 (**PSMD11**)	O00231	41.3/6.08	95	7	20		1.9	0.012
194	poly (rC) binding protein 1 (**PCBP1**)	Q15365	35.6/6.66	110	6	23		1.5	0.037
**Translation pathway**									
138	prohibitin (**PHB**)	P35232	17.4/5.57	101	5	21		−1.6	<0.001
156	ribosomal protein SA pseudogene 58 (**RPSAP58**)		29.6/9.64	126	2	17		−1.6	0.023
239	polymerase I and transcript release factor (**PTRF**)	Q6N2I2	46.2/5.51	82	5	12		1.3	0.046
**Energy metabolism**									
91	UDP-glucose 6-dehydrogenase (**UGDH**)	O60701	51.7/6.73	114	7	16		1.9	0.047
99	citrate synthase, mitochondrial (**CS**)	O75390	33.7/8.45	90	5	10		−1.9	0.021
109	isocitrate dehydrogenase 1 (NADP+), soluble (**IDH1**)	O75874	35.9/6.53	85	5	12		1.8	0.012
157	fructose-biphosphate aldolase A (**ALDOA**)	P04075	40.6/8.30	163	6	19		1.6	0.031
166	glyceraldehyde-3-phosphate dehydrogenase (**GAPDH**)	P04406	31.3/5.29	356	6	23		1.5	0.003
180	aldo-keto reductase family 1, member A1 (aldehyde reductase) (**AKR1A1**)	P14550	26.9/6.32	117	6	18		1.5	0.005
193	glucose-6-phosphate dehydrogenase (**G6PD**)	P11413	49.3/6.39	121	7	15		1.5	0.001
540	ATP synthase, H^+^ transporting, mitochondrial F1 complex, beta polypeptide (**ATP5B**)	P06576	47.1/5.26	135	9	27		−1.7	0.063
**Lipid metabolism**									
97	enoyl-CoA hydratase, short chain, 1, mitochondrial (**ECHS1**)	P30084	16.0/8.34	114	6	23		1.9	0.035
139	acetyl-CoA acetyltransferase (**ACAT2**)	Q9BWD1	28.0/6.47	107	3	11		1.6	<0.001
**Muscle development**									
111	lamin A/C (**LMNA**)	P02545	52.6/6.40	133	4	11		−1.8	0.014
126			77.8/6.40	176	12	25		1.7	0.035
451			74.2/6.40	107	7	11		1.9	0.062
**Neuronal differentiation**									
151	dihydropyrimidinase-like 2 (**DPYSL2**)	Q16555	74.2/5.85	84	5	10		1.6	0.031
483			62.2/5.85	85	6	13		1.8	0.072

Ratio values (fold change) of normalized protein spots intensities, in 2-DE gels, obtained from cells recovered from both passage 3 and passage 7 (P7/P3) after *ex-vivo* expansion, as described in the Material and Methods section.

a Estimated MW (kDa).

b Theoretical p*I.*

c Values of 0.05 or less to ensure high statistical confidence of differential expression. In some specific cases, results with ANOVA *p* values up to 0.05 (underlined) are shown.

The majority of the protein forms whose content changed at least 1.3-fold belong to the category “***Structural components and cellular cytoskeleton***” (54%) including protein members of the cellular cytoskeleton with both constitutive or modifying and rearranging functions (Table 1, Fig. 4C). The content of most of the 43 protein forms, clustered in this category, is lower in P7 compared to P3, representing 74% of all the down-regulated protein forms identified in this study. Other functional groups comprise proteins of the category “***Folding of proteins and stress response proteins***” (10%), which includes mainly heat shock proteins and proteins involved in “***Energy metabolism***” (10%). The functional group “***Apoptosis***” (6%) comprises several proteins from the Annexin family (ANXA1, ANXA2 and ANXA5) and the mitochondrial ion channel VDAC1.

Concerning cellular localization, the experimental strategy used allowed the identification of cytoplasmatic (63%), mitochondrial (16%) and plasma membrane (8%) proteins, as well as of proteins from the nucleus (8%) and endoplasmic reticulum (5%).

**Figure 5:**
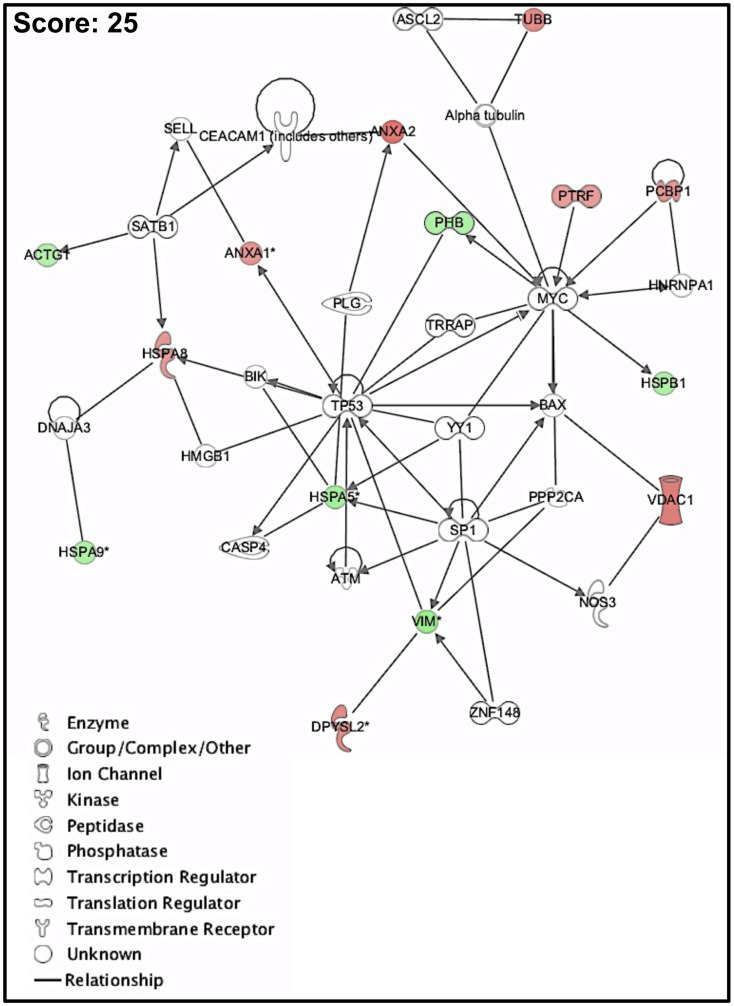
Biological network over-represented in P3 and P7 proteomes. Over-represented biological network identified by the IPA software (www.ingenuity.com). Proteins identified in the dataset are highlighted in red or in green when they exhibit a higher or a lower content in P7 compared to P3. Color intensity is directly related with protein fold change. When several protein isoforms are present, the isoform exhibiting the higher value of fold change is selected.

In order to identify the canonical pathways and the key molecular and biological functions that are over-represented within the dataset, all proteins identified whose abundance is altered in P7, compared to P3, were analyzed using the Ingenuity software (www.ingenuity.com), which is able to perform a Fischer statistical comparison between functional blocks. A dataset containing the proteins and corresponding expression values was uploaded into the application. Each protein was mapped to its corresponding object in the Ingenuity Pathways Knowledge Base. A fold change cut-off of 1.3 and *p*-value cut-off of 0.05 were set. The proteins were overlaid onto a global molecular network developed from information contained in the Ingenuity Pathways Knowledge Base. Networks of the proteins under analysis were then algorithmically generated based on their connectivity. The canonical pathways and the molecular cellular functions identified as significantly enriched (*p*≤0.001) within the dataset are summarized in Table 2. The most significant protein network identified was “*Cell death, gene expression, cellular growth and proliferation*” (score: 25) and it is represented in Fig. 5. The key nodes of this protein network are the tumor protein 53 (TP53), and the transcription factors MYC and SP1.

**Table 2:** Most significant Canonical Pathways and Molecular and Cellular Functions recognized by IPA (www.ingenuity.com), considering all the proteins whose content was altered in P7 compared to P3.

Canonical Pathways		
Pathway	*p*-value	Ratio
Protein ubiquitination pathways	5.54E-04	5/269 (0.019)
Aldosterone signaling in epithelial cells	6.67E-04	4/158 (0.025)
Caveolar-mediated endocytosis signaling	8.76E-04	3/84 (0.036)
NRF2-mediated oxidative stress response	1.17E-03	4/188 (0.021)
**Molecular and Cellular Functions**		
**Function**	***p*** **-value**	**# Molecules**
Energy metabolism	1.38E-06–4.87E-02	3
Nucleic acid metabolism	1.38E-06–4.87E-02	6
Small molecule biochemistry	1.38E-06–4.87E-02	12
Cell death	3.49E-05–3.70E-02	9

A more detailed description of the main results of the quantitative proteomic analysis performed follows.

#### Decreased abundance of proteins involved in the cytoskeleton structure and dynamics in P7 *vs* P3 cells

A total of 44 protein forms belonging to the category **“** ***Structural components and cellular cytoskeleton*** **”** were found to have a different content in P7 compared to P3 (Table 1, Fig. 4C). Of those, 24 were identified as isoforms of the protein vimentin (VIM), previously described as being expressed as different isoforms due to alternative splicing [25] or different phosphorylation states of the protein [26]. The majority of the VIM isoforms (n = 19) show a lower content in P7 compared to P3 (Table 1, Fig. 4B, 4C). VIM is the major intermediate filament protein in mesenchymal cells and it is frequently used as a developmental marker of cells and tissues [27]. This protein participates in a number of critical functions, often related to organization of proteins that are involved in adhesion, migration and cell signaling, regulation of cell death and survival, among others [27]. The regulation of the numerous protein–protein interactions and functions of VIM is presumably related with the complex phosphorylation pattern described for this protein.

In addition, multiple charge and size isoforms (n = 7) of the protein β-actin (ACTB) exhibited a lower content in P7 compared to P3 (Table 1, Fig. 4B, 4C). Together with actin gamma 1 (ACTG1), whose content is also decreased in P7 cells compared to P3, ACTB is a non-muscle type actin. The intermediate filament’s proteins keratin, type II cytoskeletal 1 (KRT1) and keratin, type I cytoskeletal 10 (KRT10), also included in the cytoskeleton category, show isoforms with a lower content in P7 compared to P3. In particular, one of the isoforms of KRT1, corresponding to the protein spot 1, exhibited the highest fold change in the dataset, (9.5x higher content in P3 compared to P7 (Table 1)). The cellular cytoskeletal composed by VIM, actin and keratins are known to be important to bridge integrin proteins with the extracellular matrix of cultured cells, making structures that play an essential role in adhesion and cell-cell interactions (reviewed in [27]). In particular, the link between actin and vimentin cytoskeleton to integrins is known to be a prerequisite for the strengthening of adhesion structures that respond to mechanical stresses [28], [29].

**Figure 6:**
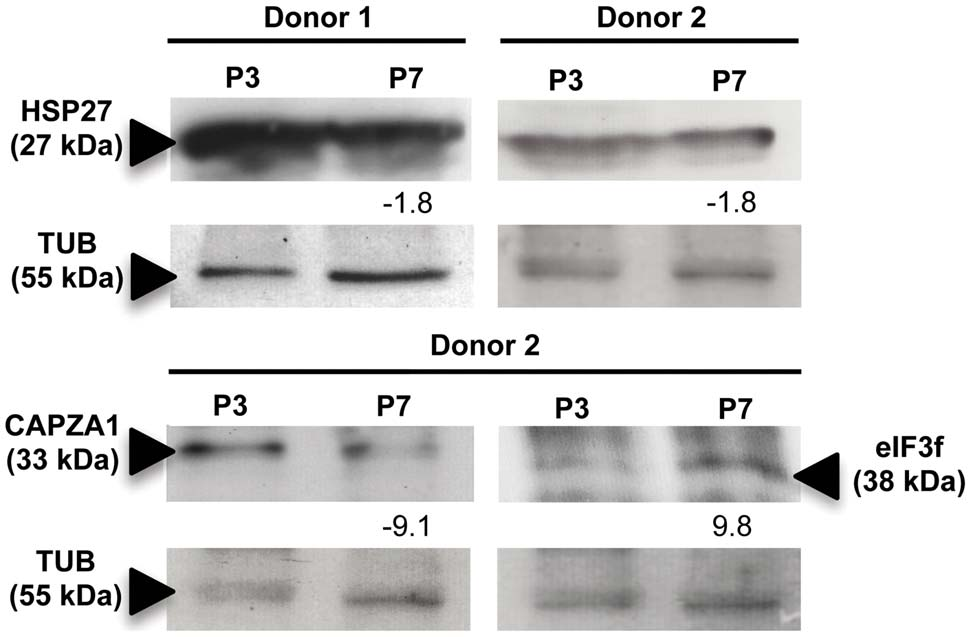
Validation of the changes in protein content of passage 7 (P7) and passage 3 (P3) cells that emerged from the quantitative proteomic analysis performed, also using cells from an additional Donor 2. Quantitative immunodetection of HSP27 (27 kDa) in Donors 1 and 2 showed a decreased content in P7 compared to P3 (upper panel). CAPZA1 (33 kDa) and eIF3f (38 kDa) exhibited decreased or increased levels, respectively, in P7 cells compared to P3, in Donor 2 (lower panel). All these results are consistent with the indications from 2-DE expression levels obtained for Donor 1. The values shown are the fold changes in P7 *vs.* P3, as measured by densitometry and normalized using the TUB (55 kDa) levels as the internal control.

The decreased content of the protein capping protein (actin filament) muscle Z-line, alpha 1 (CAPZA1; 33 kDa) in P7 compared to P3 (Fig. 6) revealed by quantitative proteomic analysis performed for Donor 1 (Table 1) was validated by quantitative immunodetection for an additional donor (Donor 2).

#### Decreased abundance of chaperone proteins and stress response proteins in P7 *vs.* P3 cell proteomes

The extended *ex-vivo* cultivation of BM MSC resulted in a decreased content of proteins of the stress response, in particular heat shock proteins (Table 1, Fig. 4C). Specifically, the proteins whose content decreased in P7 compared to P3 include the 78 kDa glucose-regulated protein HSPA5 or BiP, the heat shock 70 kDa protein 9 HSPA9 or HSP70, the heat shock protein beta-1 HSPB1 or HSP27 and the protein disulfide-isomerase (P4HB). Quantitative immunodetection confirmed the decreased content of HSP27 (Table 1) in P7 compared with P3 cells obtained from Donor 1 and Donor 2 (Fig. 6). Chaperone proteins are known to contribute to cell redox homeostasis by preventing the aggregation of damaged or unfolded proteins, protecting cells from entering into apoptosis [30]. Differently, the content of a protein form identified as HSPA8 or HSC70 (spot 207), was higher in P3 compared to P7 (Table 1, Fig. 4C). This protein is, presumably, a shorter HSPA8 splice variant with 54 kDa, HSC54, resulting from an alternate in-frame splice site in the 3′ coding region. HSC54 was proposed to be an inhibitory regulator of HSPA8 [31], involved in protein folding of newly synthesized polypeptides, translocation of proteins across the endoplasmic reticulum, stabilization of proteins under stress conditions, antigen presentation and endocytosis [32].

#### Increased expression of Apoptosis-related proteins in P7 vs. P3 cells

Results also show an increased content of a number of apoptosis related proteins in the later passage studied compared to P3: annexin A1 (ANXA1), A2 (ANXA2) and A5 (ANXA5) and the voltage-dependent anion-selective channel protein 1 (VDAC1) (Table 1, Fig. 4C). The overexpression of ANXA2 has been related with a reduction in cell proliferation what is consistent with the lower proliferative capacity of P7 cells, compared to P3 described herein, and the protein ANXA2 was previously attributed to the aging process of the organism and replicative senescence [11], [33]. Under apoptotic signals, VDAC1 leads to increasing mitochondrial membrane permeability, resulting in the release of small molecules such as cytochrome c, Smac/Diablo and AIF [34].

The content of the transcriptional regulator prohibitin (PHB), clustered into the category “*Translation*”, exhibits a lower content in P7 compared to P3 (Table 1). This protein has a role in cell cycle control, differentiation, senescence and anti-proliferative activity [35], [36] and PHB expression was suggested to prevent cells to undergo apoptosis [37].

The protein glyceraldehyde-3-phosphate dehydrogenase (GAPDH), a pivotal protein in energy production, exhibits higher content levels in P7 compared to P3 (Table 1). Moreover, GAPDH also plays a critical role in triggering apoptosis by the induction of pro-apoptotic proteins under age-induced stimulus, such as VDAC1 [38], [39].

#### Increased content of proteins involved in cell cycle regulation and aging in P7 *vs.* P3 cells

All the proteins belonging to the category “***Cell cycle regulation and aging***” found in this study (Table 1) are more abundant in cells from passage 7 compared to passage 3 cells. Among those proteins, the eukaryotic translation initiation factor 3, subunit F (eIF3f), was found to have a markedly increase (3.1-fold) in P7 compared to P3. This increased content of the protein eIF3F in P7 compared to P3, for Donor 1 revealed by this quantitative proteomic analysis (Table 1), was validated by quantitative immunodetection using cells from Donor 2 (Fig. 6).

The proteasome 26S subunit, non-ATPase, 11 protein (PSMD11) exhibits a 1.9x higher content in P7 compared to P3 (Table 1). PSMD11 is a non-ATPase subunit belonging to the 19S regulator of the 26S proteasome complex, known to be involved in the unfolded protein response (UPR), an important genomic response to endoplasmic reticulum stress [40]. During UPR, accumulated unfolded proteins are either correctly refolded or unsuccessfully refolded and degraded in the cytosol by the 26S proteosome, via the ubiquitin-proteasome system, allowing a tight control of critical cellular functions such as DNA repair, cell cycle progression, development, apoptosis, gene transcription, signal transduction, senescence, immune response, metabolism and protein quality control. Consistent with the increased content of PSMD11 in P7 cells compared to P3 cells, the “***protein ubiquitination pathway***” is the most represented canonical pathway altered (Table 2) as a consequence of consecutive passaging of BM MSC in culture.

#### Increased content of proteins involved in energy and lipid metabolism in P7 *vs.* P3 cells

The functional category “***Energy metabolism***” includes 6 proteins whose content is higher in P7 compared to P3 (Table 1, Fig. 4C) and, as revealed by IPA software (www.ingenuity.com), it appears as the most represented altered molecular function among the proteins whose content was different in P7 compared to P3 (Table 2). The proteins belonging to this category are involved in several metabolic pathways for ATP or NADPH synthesis such as glycolysis (fructose-biphosphate aldolase A, ALDOA; aldo-keto reductase family 1, member A1, AKR1A1); glyceraldehyde-3-phosphate, GAPDH), the tricarboxylic acid cycle (TCA cycle) (isocitrate dehydrogenase 1, IDH1), the pentose phosphate pathway (glucose-6-phosphate dehydrogenase, G6PD) and the biosynthesis of UDP-glucose in the glucuronic acid biosynthetic pathway (UDP-glucose 6-dehydrogenase, UGDH).

Two proteins of the category “***Lipid metabolism***”, enoyl-CoA hydratase, short chain, 1 (ECHS1) and acetyl-CoA acetyltransferase (ACAT2), known to be involved in the synthesis of acetyl-CoA and pivotal for the TCA cycle, were also found to have a higher content in P7 compared to P3.

## Discussion

Mesenchymal stem cells-based therapies rely largely on the preparation of an effective dose of *ex-vivo* expanded cells obtained by consecutive cell passaging during long-term cultivation. However, the effects of extended *ex-vivo* cultivation ultimately may lead to a senescent state of the cultured cells and although several biological processes have been described to be involved in the long-term culture effects on BM MSC populations [23], the precise molecular profile of MSC is still unknown, turning the definition of reliable molecular targets indicators of cellular senescence extremely needed.

In our study, we observed that the sequential *ex-vivo* cell passaging, since P1 until P9, of BM MSC results in a progressive loss of the proliferative and clonogenic potential, a progressive alteration in cell morphology (cells became bigger and with a more granular cytoplasm) and a slight decrease of the expression of CD73 and CD105, in line with previous studies [11]. The reduction in telomere length, an evident marker for cell senescence, in P7 compared to P3 is also likely, considering the significant reduction of telomere length (22.5%), registered before after 3 passages (P3 to P6) of BM MSC grown under the conditions used in the present work [16].

To gain mechanistic insights into the replicative senescence phenomenon occurring along BM MSC consecutive passage cultivation and to contribute to the development of markers for the early detection of *ex-vivo* cellular senescence when no significant differences are still observed at the cellular level, we compared the proteomic expression profile of cells retrieved from the passage 3 (P3), commonly used in clinical studies with expanded MSC, and passage 7 (P7). Although some studies may consider P7 as a low passage (less than 20 cumulative population doublings), taking into account our experimental data, this passage already demonstrates significant signs of culture-induced senescence. In the literature, higher passages, namely P8 or P12, have been considered as reference for “late” passages for which the occurrence of replicative senescence was clearly demonstrated based on mRNA and miRNA profiling [11], [23].

Although an expression proteomic analysis based on 2-DE cannot assess the alterations registered in the expression of all the genes in the genome, as it is virtually possible at least at the transcriptional level based on a pangenomic DNA microarrays [11], [23], this genome-wide expression analysis offers a chance to identify post-transcriptional modifications. Indeed, in this study, it was possible to identify a large number of isoforms of different proteins whose content was altered in P7 *vs.* P3, reinforcing the concept that post-transcription and post-translational modifications should be considered among the mechanisms underlying the control of the expression programs of stem cell populations under extended cultivation, which is a step forward in the understanding of the mechanisms behind replicative senescence of BM MSC [15], [41].

The markedly decrease in the content of protein forms involved in cytoskeleton structure and dynamics observed in P7 cells, compared to P3 cells, suggests that the *ex-vivo* cultivation of MSC along consecutive passages leads to a senescent stage characterized by impaired cytoskeleton remodeling and/or organization capacity, consistent with the observed loss of MSC proliferative and clonogenic potential. It is interesting to note that the aging phenomenon is responsible for MSC proteome alteration at the level of cytoskeleton organization, antioxidant defenses, and actin dynamics, and decreased actin turnover [42].

Most of the proteins involved in stress response, namely the heat shock proteins HSPA5, HSPA9 and HSPB1, show a lower content in cells recovered from a higher senescence level (P7) compared to the early passage (P3). The down-regulation or inhibition of HSPB1 or HSPA9 have been shown to be enough to sensitize a cell to apoptosis [43]. The HSPA9 content was also previously found to decline when cells approach senescence [44] and the down-regulation of HSPB1 was described, in part, as responsible for the induction of senescence by activation of p53 and induction of p21, the major regulators of the senescence program [45]. Moreover, HSPB1 is also known to inhibit the cytochrome-c-mediated activation of caspases in the cytosol, preventing the entry into apoptosis [46]. On the other hand, HSPA5 chaperone, together with P4HB whose content was also lower (over 2-fold) in P7 cells compared to P3, stimulates protein refolding and degradation [47]. HSPA5 was also described as a central regulator in the UPR [40]. The higher content of proteins involved in protein degradation (PSMD11) and translation initiation (eIF3F) in P7, compared to P3, suggests the occurrence of increased protein degradation by the 26S proteasome, a mechanism that has high-energy requirements [48]. The protein eIF3f is the p47 subunit of the eIF3 complex which plays an important role in translation initiation [49] and whose over-expression, in tumor cells, inhibits cell proliferation and induces apoptosis [50]. BM MSC consecutive passaging is expected to lead to an accumulation of protein aggregates containing aberrantly folded proteins as a result of the stress induced during long-term cultivation, promoting senescence and potentially leading to apoptosis [51]. Consistently, many reports in the scientific literature document a gradual decline in the repair and maintenance systems in aging cells [44], which leads to age-related cellular dysfunction [52]. Taken together, the decrease of the content of proteins required for protein folding and the increased content of proteins involved in protein degradation and apoptosis suggest an impaired cellular response of P7 cells (compared to P3) for trafficking, repair and rapid elimination of misfolded proteins that might underlie the progressive loss of the proliferative and clonogenic features of expanded BM MSC and trigger the mechanisms that will lead to apoptosis. Moreover, the observed increase of the content of several proteins involved in “***Energy metabolism***” is consistent with cell adaptation to the energetic demands related with protein degradation mechanisms and apoptosis.

The higher content of several proteins involved in apoptosis, such as ANXA1, ANXA2, ANXA5 and VDAC1 is consistent with the suggested increased level of apoptosis in P7 cells, compared to P3. Moreover, beside the role of ANXA1 in apoptosis, this protein was also described as having an anti-proliferative function, namely by the disruption of the actin cytoskeleton [53]. Interestingly, although at the proteome level this study suggests a higher incidence of apoptosis in P7 cells, no significant differences in the number of apoptotic cells were observed for P3 and P7 cultures when the Annexin V Staining Protocol kit by flow cytometry was used [54] (0.82% and 0.93%, respectively (unpublished results)), reinforcing the idea that development of more sensitive methodologies is needed to comprehensively unveil targeted cellular processes.

The impact of *ex-vivo* cultivation in the induction of a senescence state is likely to be also related to exposure to the stressing culture environment itself, including the basal medium and fetal bovine serum used and the atmospheric oxygen tension, among other factors, that largely differ from the microenvironment where these cells reside *in vivo*. In this context, we have recently reported the beneficial effects of cultivating human MSC in a low oxygen environment (2%), more closely to the *in vivo* BM hypoxic niche, namely by promoting a faster cell proliferation kinetics and a more efficient metabolism [16]. In addition, consecutive passaging requires the use of enzymatic reagents such as Accutase® (used herein) or trypsin at cell sub-confluency in order to harvest the cells and re-seed those into new culture flasks. The use of such proteolytic agents is also a source of stress to the cells since many proteins located on the cell surface are often cleaved, leading to dysregulation of cellular functions. Indeed, the trypsin treatment has been reported as having effects on cell shape and chromatin structure of animal cells [55] as well as in the proteomic profiling, namely decreasing the content of proteins involved in cell metabolism and growth regulation, mitochondrial electron transport and cell adhesion and increasing the content of apoptosis-related proteins [56]. Although the enzymatic agent used in our studies - Accutase® - is known to gently and effectively detach adherent cells as human MSC, its proteolytic and collagenolytic activity might have affected the cytoskeleton structure and specific membrane proteins such as chaperones known to be highly abundant on the cell surface [57].

Based on the use of GeneCoDis software [58], [59] it is possible to hypothesize the most significant regulatory pathways that may underlie the coordinate expression regulation of the proteins whose content was altered in P7 compared to P3 (Table 3). The transcription factor MAZ (Myc-associated zinc-finger protein) is suggested to regulate 10 proteins from the dataset while the transcription factors AP1 and SP1 are suggested to bind to the promoters of four and seven of these genes, respectively, found to encode proteins whose content is also altered. The zinc finger transcription factor MAZ is known to activate the expression of tissue-specific genes and to repress the expression of the c-myc proto-oncogene that codes for the protein MYC [60] while the transcription factors MYC, AP1 and SP1 are known to be involved in the regulation of proliferation, differentiation and apoptosis pathways [61], [62]. Remarkably, the expression of VIM protein is suggested to be co-regulated by MAZ, AP1 and SP1, and several other proteins are putatively regulated by two of these three transcription factors (Table 3). These are interesting indications that provide guidance for in-depth studies on the regulation of the expression program of human MSC upon extended *ex-vivo* cultivation.

**Table 3:** The most significant transcriptional regulators recognized by the GeneCoDis 2 software [58], [59].

Transcription factor	Genes	Hyp^a^
**MAZ**	**HSPA8**, **ALDOA**, LMNA, **PTRF**, **IDH1**, ACTB, **CS**, VIM, **CAPG**, HSPB1	9.51E-05
**AP1**	**ALDOA**, LMNA, **PSMD11**, VIM	1.66E-04
**SP1**	HSPA5, **IDH1**, **UGDH**, **CS**, VIM, **DPYSL2**, HSPB1	6.27E-04

a hypergeometric *p*-value.

The regulated proteins whose content is higher in P7, compared to P3, are in bold, proteins whose content is lower in P7 are in regular while proteins presenting several isoforms with different expression values are underlined.

The manufacture of a reliable and safe cell product is nowadays critical to guarantee the continuous advances of MSC-based therapies. Independently of the culture system used for MSC expansion, cell senescence caused by intensive cell proliferation is a major concern, as it may jeopardize the treatment efficacy. In this study we showed that although after 7 passages BM MSC still maintained their characteristic features (adherence to plastic, immunophenotype and multilineage differentiation potential), the proliferative and clonogenic potential were seriously affected, as well as their proteome profile, namely for proteins in the categories “***Structural components and cellular cytoskeleton***”, “***Folding and stress response proteins***”, “***Energy metabolism***”, “***Cell cycle regulation and aging***” and ***“Apoptosis***”. Since the approval of stem cell-based therapies by regulatory agencies (EMA or FDA) relies on a full characterization and safety of the cellular product, our study is a contribution to the efforts being made in this field, paving the way to the establishment of a proteomic analysis platform as a quality control panel for cultured MSC.

## Materials and Methods

### Human Donor Sample, Cell Culture and Characterization

#### MSC isolation and cell propagation

Bone marrow (BM) aspirates were obtained from four healthy adult donors (Donors 1–4; average age of 42±2 (minimum of 39 and maximum of 44)) after written informed consent and the procedure was approved by the Ethics Committee of Instituto Português de Oncologia Francisco Gentil, Lisboa, Portugal. Mesenchymal stem cells (MSC) were isolated according to the protocol described elsewhere [16]. Upon isolation (Passage 0 (P0) –7 days in culture), monolayers were washed with Phosphate Buffered Saline (PBS) (GibcoBRL, Grand Island, NY, USA) and detached from the culture flasks by adding Accutase® solution (Sigma-Aldrich, St. Louis, MO, USA) for 7 minutes at 37°C. Then, cells were washed twice with PBS and collected by centrifugation at 200×g during 7 minutes. Cell number and viability were determined using the Trypan Blue Stain 0.4% solution (GibcoBRL) and then cells were replated at an initial density of 3000 cells/cm^2^ in T-175 flasks (BD Falcon, Franklin Lakes, NJ, USA) using Dulbecco’s Modified Essential Medium-Low Glucose (DMEM-LG, Gibco, Carlsbad, California) with 10% Fetal Bovine Serum (FBS, MSC qualified, GibcoBRL). After 7 days of culture (P1), MSC were cryopreserved at an average cell density of 5×10^5^ cells/mL in CryoTube™ vials (Nunc, Roskilde, Denmark). Cells were centrifuged and resuspended in Recovery™ Cell Culture Freezing Medium (Invitrogen, Carlsbad, CA) in 1 mL cryovials, which were kept for 24 hours in a −80°C freezer before being stored in liquid nitrogen (−196°C) until further use. When needed, MSC were thawed at 37°C and resuspended in Iscove’s Modified Eagle’s Medium (IMDM) (GibcoBRL) supplemented with 20% FBS. Cell viability was checked using the Trypan Blue test and was always higher than 95%. Cells were then centrifuged at 200×g for 7 minutes and resuspended in culture medium (DMEM-LG+10%FBS) and plated at an initial density of 3000 cells/cm^2^ in T-75 flasks (P2). This protocol was followed for the consecutive passages (up to passage 9).

#### Proliferative analysis

During time in culture, the *ex-vivo* expansion of the BM MSC was determined by using the Trypan Blue exclusion method. Cell proliferation along passages was studied by determining the fold increase in total cell number, which is calculated by dividing the number of cells at day 0. Specific growth rates (µ, day^−1^) were determined for each passage as previously described [16]. Population doublings were also calculated for every passage by dividing the logarithm of the fold increase value obtained at the end of the passage by the logarithm of 2.

#### Clonogenic ability assays

Colony forming units-fibroblast (CFU-F) assays were performed at day 7 as previously described [16]. Briefly, cultured cells were plated at a cell density of 10 cells/cm^2^ and kept at 37°C, 5% CO_2_ in a humidified incubator for 14 days. Cells were then washed once with PBS and incubated with a 0.5% crystal violet (Sigma-Aldrich) solution (30 minutes). Finally, stained colonies were rinsed 4 times with PBS and then with distilled water. After drying, colonies with 50 or more cells were counted.

### Multilineage Differentiation Assays

#### Adipogenic differentiation

BM MSC were plated at 3000 cells/cm^2^ on 12-well plates and adipogenic differentiation was induced at 80% cell confluence after culture for 14 days, using StemPro® Adipogenesis Differentiation Kit (Invitrogen). The medium was changed twice a week for 14 days. The assessment of differentiation towards an adipocytic phenotype was performed based on the accumulation of lipids, using Oil Red-O stain. Cells were washed with cold PBS and fixed in 2% formaldehyde for 30 minutes. After fixation, cells were then washed with distilled water and incubated with Oil Red-O solution (Sigma-Aldrich) (0.3% in isopropanol) at room temperature for 1 hour. Finally, cells were washed with distilled water and observed under the microscope (Leica Microsystems, Wetzlar, Germany).

#### Osteogenic differentiation

BM MSC were plated at 3000 cells/cm^2^ on 12-well plates and at 80% cell confluency, osteogenesis was induced using StemPro® Osteogenesis Differentation Kit (Invitrogen). The medium was changed twice a week for 14 days. After induction, cells were prepared for alkaline phosphatase (ALP) and von Kossa staining. Briefly, cells were washed in cold PBS and fixed in 10% cold neutral-buffered formalin (Sigma-Aldrich) for 15 minutes. After fixing, cells were washed and kept in distilled water for 15 minutes. Cells were incubated with a 0.1 M solution of Tris-HCl (Sigma-Aldrich) containing Naphtol AS MX-PO4 (0.27 mM) (Sigma-Aldrich) in dimethylformamide (Fischer Scientific, Pittsburgh, PA, USA) and 1.59 mM of Red Violet LB salt (Sigma-Aldrich) for 45 minutes and washed 4 times with distilled water. Cells were then observed under the microscope for ALP staining, as a result of osteogenic commitment. Cells were then stained with silver nitrate (2.5% w/v) (Sigma-Aldrich) for 30 minutes at room temperature for von Kossa staining to evaluate the deposits of calcium in the cultures. Cells were washed 3 times in distilled water, set to dry and observed in the microscope.

#### Chondrogenic differentiation

Expanded BM MSC were plated as small droplets (5–10 µL) with high cell densities on ultra low attachment culture plates (Corning, Lowell, MA, USA). After 30 minutes, StemPro® Chondrogenesis Differentation Kit (Invitrogen) was added. The medium was changed twice a week for 14 days. The assessment of differentiation towards a chondrocytic phenotype was performed based on the synthesis of proteoglycans by chondrocytes, using Alcian Blue stain. Cells were washed with cold PBS and fixed in 2% formaldehyde for 30 minutes. After fixation, cells were then washed with distilled water and incubated with 1% Alcian Blue solution (Sigma-Aldrich) at room temperature for 1 hour. Finally, cells were washed with distilled water and observed under the microscope.

### Immunophenotypic Analysis

At passages 3 (P3) and 7 (P7) (upon 7 and 35 days of culture after thawing, respectively), cultured cells were analyzed by flow cytometry FACSCalibur equipment, (Becton Dickinson, Franklin Lakes, NJ, USA). The characteristic immunophenotype of human BM MSC include the expression of CD73, CD90 and CD105 (>90%). A panel of mouse anti-human monoclonal antibodies (PE-conjugated) against: CD73 (Becton Dickinson Immunocytometry Systems), CD90 (R&D Systems, Minneapolis, USA) and CD105 (Invitrogen) was used. Cells were incubated with these monoclonal antibodies for 15 minutes in the dark at room temperature and then cells were washed in PBS and fixed with 1% paraformaldehyde (Sigma-Aldrich). Isotype controls were also prepared for every experiment. A minimum of 10000 events was collected for each sample and the CellQuest software (Becton Dickinson) was used for acquisition and analysis [16].

### Statistical Analysis

When appropriate, comparisons between experimental results were determined by the non-parametric Mann-Whitney U test. A *p*-value less than 0.05 was considered statistically significant.

### 2-DE Quantitative Proteomic Analysis

#### Preparation of protein samples

To generate the 2-D map of MSC and analyze BM MSC protein changes during long-term *ex-vivo* culture, cells from Donor 1 (male, 44 years old) from two different cell passages were chosen, Passage 3 (P3) and Passage 7 (P7) and processed for protein extraction. Each biological sample (per passage) was prepared by pooling together cell samples obtained from three independent cultures prepared from BM MSC at P1 (see 4.1.1). Cells were washed with PBS and harvested from the culture flasks by adding Accutase® solution as previously described. The cell pellets containing around 3×10^6^ cells were resuspended in 500 µL Lysis buffer (8M urea, 4% (w/v) CHAPS and traces of bromophenol blue supplemented with 15 mM DTT, 0.5% (v/v) pharmalytes 3–10 (Amersham Biosciences, Uppsala, Sweden) and 100 mM sucrose Halt™ protease inhibitors cocktail (Pierce, Rockford, USA)). The mixture was homogenized by sonication on ice and left to stand at room temperature for 2 hours. Cell debris were pelleted down by centrifugation at 6000 x g, 4°C, during 10 minutes and the supernatant was transferred to a clear microcentrifuge tube. Protein concentration of both protein samples was quantified using 2-D Quant kit (GE Healthcare, Piscataway, NJ, USA). To eliminate any contaminant that may interfere with the subsequent 2-DE procedure, 100 µg aliquots of the protein samples were subjected to a clean-up process, using the 2-D Clean-up kit (GE Healthcare). The internal standard sample was prepared by using a mix of 50 µg of each protein sample (from P3 and P7 cells).

#### Proteome separation

For the isoelectric focusing (IEF) of the protein samples, Immobiline DryStrips with 24 cm and a 3–10 non-linear pH range (GE Healthcare) were rehydrated overnight in 450 µL of reswelling buffer (7 M urea, 2 M thiourea, 2% (w/v) CHAPS, 0.002% (w/v) bromophenol blue, 1.2% (v/v) DeStreak (GE Healthcare) and 0.5% (v/v) pharmalytes (GE Healthcare)). Two replicates of each sample containing proteins from P3, P7 or from the internal standard samples were gently resuspended in 80 µL of IEF buffer (7 M urea, 2 M thiourea, 2% (w/v) CHAPS, 0.002% (w/v) bromophenol blue, 15 mM DTT and 2% (v/v) pharmalytes 3–10 (GE Healthcare)), prior to anodic cup loading on the Immobiline DryStrips. Preparative gels for protein identification were prepared using 300 µg of an internal standard sample (mix of 150 µg of each P3 and P7 samples). IEF was performed using an Ettan IPGphor instrument (GE Healthcare), as previously described [21]. After IEF, strips were equilibrated for 15 minutes using 4 mL of equilibration buffer containing 6 M urea, 30% (v/v) glycerol, 2% (w/v) SDS, 50 mM Tris at pH 8.8, 0.002% (w/v) bromophenol blue and 1% (w/v) DTT, followed by a second incubation period of 15 minutes with the same buffer containing 3% (w/v) iodoacetamide. Then, separation of the proteins according to their molecular weight (MW) was performed on 24 cm 12% SDS-polyacrylamide gels, using the EttanDALTsix instrument (GE Healthcare) [21], [22]. After gel labeling with the fluorescent dye Flamingo (Bio-Rad) along 15 hours in a 1∶10 dilution, analytical gels were scanned on a Typhoon Trio laser scanner (GE Healthcare) and preparative gels containing 300 µg were stained with silver nitrate.

#### Gel image analysis

Analytical gel images were analyzed with Progenesis Samespots software package (Nonlinear Dynamics, Newcastle, UK), as described elsewhere [20]. Protein spots were identified using the automatic spot detection algorithm. Individual spot volumes were normalized against total spot volumes for a given gel. Averages values for each passage sample were then compared by their normalized volume using one-way ANOVA between-group test. Only statistically significant spots (*p*<0.05) were selected for analysis, unless otherwise stated. Differential expression between the two passages was quantified and a threshold of at least 1.3-fold increase or decrease between averaged gels was considered.

#### Protein identification and data analysis

The identification of protein spots of interest was performed by mass spectrometry at the proteomic unit at Centro Nacional de Investigaciones Cardiovasculares Carlos III, Madrid, Spain (CNIC Foundation), as described before [20], [22]. Briefly, protein spots were excised manually from polyacrylamide gels and then digested automatically using a Proteineer DP protein digestion station (Bruker-Daltonics, Bremen, Germany). The digestion protocol used was that of Shevchenko *et al* [63] with minor variations: gel plugs were submitted to reduction with 10 mM dithiothreitol (GE Healthcare) in 50 mM ammonium bicarbonate (99.5% purity; Sigma Chemical, St. Louis, MO, USA) and alkylation with 55 mM iodoacetamide (Sigma Chemical, Steinheim, Germany) in 50 mM ammonium bicarbonate. The gel pieces were then rinsed with 50 mM ammonium bicarbonate and acetonitrile (gradient grade; Merck, Darmstadt, Germany) and dried under a stream of nitrogen. Modified porcine trypsin (sequencing grade; Promega, Madison, WI, USA) at a final concentration of 13 ng/µL in 50 mM ammonium bicarbonate was added to the dry gel pieces and the digestion proceeded at 37°C for 6 hours. Finally, 0.5% (v/v) trifluoroacetic acid (99.5% purity; Sigma Chemical) was added for peptide extraction. An aliquot of the above digestion solution was mixed with an aliquot of α-cyano-4-hydroxycinnamic acid (Bruker-Daltonics) in 33% (v/v) aqueous acetonitrile and 0.1% (v/v) trifluoroacetic acid. This mixture was deposited onto a 600 µm AnchorChip MALDI probe (Bruker-Daltonics) and allowed to dry at room temperature. MALDI-MS(/MS) data were obtained using an Ultraflex time-of-flight mass spectrometer (Bruker-Daltonics) equipped with a LIFT-MS/MS device [64]. Spectra were acquired in the positive-ion mode at 50 Hz laser frequency, and 100 to 1500 individual spectra were averaged. For fragment ion analysis in the tandem time-of-flight (TOF/TOF) mode, precursors were accelerated to 8 kV and selected in a timed ion gate. Fragment ions generated by laser-induced decomposition of the precursor were further accelerated by 19 kV in the LIFT cell and their masses were analyzed after passing the ion reflector. Measurements were in part performed using post- LIFT metastable suppression, which allowed removal of precursor and metastable ion signals produced after extraction out of the second ion source. Detailed analysis of peptide mass mapping data was performed using flexAnalysis software (Bruker-Daltonics). Internal calibration of MALDI-TOF mass spectra was performed using two trypsin autolysis ions with m/z = 842.510 and m/z = 2211.105; for MALDI-MS/MS, calibrations were performed with fragment ion spectra obtained for the proton adducts of a peptide mixture covering the 800–3200 m/z region. MALDI-MS and MS/MS data were combined through MS BioTools program (Bruker-Daltonics) to search the NCBInr database using Mascot software (Matrix Science, London, UK) [65]. All the proteins identified are listed in Table S1 of the Supplementary Material and in Table 1.

#### Biological pathway profiling

In order to guide the proposal of the putative function of each protein mapped in the 2-DE gels and whose content is altered in the two passages under study, the biological pathway profiling was performed using the Uniprot database (http://www.ebi.uniprot.org/index.shtml), the Human Protein Atlas (http://www.proteinatlas.org7index.php) and the Human Genome Resources gateway (http://www.ncbi.nlm.nih.gov/genome/guide/human/). Proteins showing altered abundance in P7 in comparison to P3 were further analyzed for enrichment of specific biological pathways using the web-based tool GeneCoDis 2 (http://genecodis.dacya.ucm.es/) [58], [59] using the hypergeometric distribution p-values as performed by the software, with *p*≤0.001 considered significant. The determination of the most relevant molecular networks and biological pathways was generated by IPA (Ingenuity Systems, www.ingenuity.com).

### Western-Blotting

Total protein lysates (20 µg) from BM MSC cultures at passages 3 and 7 both from Donor 1 (the one examined by quantitative proteomic analysis) and Donor 2 (male, 43 years old) were separated in a 12% SDS-PAGE gel and transferred to a nitrocellulose membrane (PALL Life Sciences, BioTrace™ NT, Pensacola, Florida). The blots were blocked with Tris-buffered saline containing 0.1% Tween-20 and 5% non-fat milk, followed by incubation with goat anti-human primary antibodies against the heat shock protein 27 (HSP27), the capping protein (actin filament) muscle Z-line, alpha 1 (CAPZA1), the eukaryotic translation initiation factor, subunit f (eIF3f) and the α-tubulin (TUB) (all the primary antibodies were from Santa Cruz Biotechnology, Inc., Santa Cruz, California). The bound primary antibodies were detected by using a mouse anti-goat IgG-horseradish peroxidase conjugated antibody (Santa Cruz Biotechnology, Inc.) for all the tested proteins. TUB was used as the internal control. The Western blots were scanned and analyzed by densitometry using the ImageJ software. Normalization was performed using the TUB expression levels detected.

## Supporting Information

Table S1 **Proteins identified in this work by mass spectrometry.** For each protein, the accession number, theoretical p*I* and molecular weight are indicated. The protein identification was obtained by mass spectrometry.
(PDF)
