# Supplementary material for: Human Mesenchymal Stem Cell Expression Program upon Extended Ex-Vivo Cultivation, as Revealed by 2-DE-Based Quantitative Proteomics
Source: PLoS One. 2012 Aug 20;7(8):e43523. doi: 10.1371/journal.pone.0043523 (PMC3423346; doi:10.1371/journal.pone.0043523)
Supplement: Table S1 — Proteins identified in this work by mass spectrometry. For each protein, the accession number, theoretical pI and molecular weight are indicated. The protein identification was obtained by mass spectrometry. (PDF) [file pone.0043523.s001.pdf]

SUPPLEMENTARY MATERIAL

This document contains:

Table S1 – Proteins identified in this work by mass spectrometry.

Table S1 – Proteins identified in this work. For each protein, the accession number, theoretical p*I* and molecular weight are indicated. The protein identification was obtained by mass spectrometry.

Spot (nr.)	Protein function	Accession Number	MW (KDa)/ p*I*	Mascot score	No. of matched peptides	Sequence coverage (%)
**Structural components and cellular cytoskeleton**						
3	actin, cytoplasmic 1; beta-actin (**ACTB**)	P60709	42.1/5.29	243	5	18
7			42.1/5.29	244	6	21
35			42.1/5.29	144	4	13
64			42.1/5.29	158	3	12
81			42.1/5.29	116	4	13
160			42.1/5.29	356	15	42
171			42.1/5.29	97	5	18
1	keratin, type II cytoskeletal 1 (**KRT1**)	P04264	66.1/8.15	154	12	24
59			66.1/8.15	96	8	17
75			66.1/8.15	58	5	9
158			66.1/8.15	91	8	15
148	keratin, type I cytoskeletal 10 (**KRT10**)	P13645	59.0/5.13	85	7	10
178			59.0/5.13	95	8	18
33	vimentin (**VIM**)	P08670	53.7/5.06	128	7	23
39			53.7/5.06	277	17	29
41			53.7/5.06	249	16	44
55			53.7/5.06	497	21	41
56			53.7/5.06	258	13	34
58			53.7/5.06	403	24	49
67			53.7/5.06	294	16	39
74			53.7/5.06	227	15	37
78			53.7/5.06	406	22	34
79			53.7/5.06	204	9	21
87			53.7/5.06	82	3	7
106			53.7/5.06	173	13	29
113			53.7/5.06	248	12	27
124			53.7/5.06	160	10	30
128			53.7/5.06	103	6	16
131			53.7/5.06	400	18	39

Spot (nr.)	Protein function	Accession Number	MW (KDa)/ p*I*	Mascot score	No. of matched peptides	Sequence coverage (%)
202			53.7/5.06	164	9	27
484			53.7/5.06	318	20	45
603			53.7/5.06	713	33	63
98			53.7/5.06	157	15	46
121			53.7/5.06	192	11	29
146			53.7/5.06	157	9	24
198			53.7/5.06	883	29	59
236			53.7/5.06	101	6	16
353			35.1/4.7	225	9	37
795			53.7/5.06	414	24	57
849			53.7/5.06	279	20	48
879			53.7/5.06	288	16	35
20	macrophage-capping protein (**CAPG**)	P40121	38.8/5.88	158	6	23
53	fascin homolog 1, actin bundling protein (*Strongylocentrotus purpuratus*) (**FSCN1**)	Q16658	55.1/6.84	121	7	17
77	capping protein (actin filament) muscle Z-line, alpha 1 (**CAPZA1**)	P52907	33.1/5.45	115	6	24
102	tubulin beta chain (**TUBB**)	P07437	40.6/4.78	161	9	19
183	actin, gamma 1 (**ACTG1**)	P63261	26.1/5.65	136	3	16
208	twinfilin, actin binding protein (**TWF1**)	Q12792	39.8/6.37	149	4	20
**Folding of proteins and stress response proteins**						
60	protein disulfide-isomerase (P55) (**P4HB**)	P07237	57.4/4.76	89	6	11
85	78 kDa glucose-regulated protein (**HSPA5**)	P11021	72.4/5.07	266	7	14
529			72.4/5.07	243	10	18
242	heat shock 70 kDa protein 9 (mortalin) (**HSPA9**)	P38646	73.9/5.87	166	7	13
581			73.9/5.87	111	7	15
144	heat shock protein beta-1 (HSP 27) (**HSPB1**)	P04792	22.8/5.98	113	3	20
176	peptidyl-prolyl cis-trans isomerase B (cyclophilin B) (**PPIB**)	P23284	23.7/9.42	93	6	30
207	heat shock cognate 71 kDa protein (**HSPA8**)	P11142	71.1/5.37	227	9	18
254	endoplasmic reticulum protein ERp29 (**ERP29**)	P30040	29.0/6.77	126	4	19
**Apoptosis**						
168	annexin A1 (**ANXA1**)	P04083	38.9/6.57	351	14	49
698			38.9/6.57	307	17	50
364	annexin A2 (**ANXA2**)	P07355	38.8/7.57	114	6	21
620			38.8/7.57	143	8	25
494	annexin A5 (**ANXA5**)	P08758	36.0/4.94	121	6	20
45	voltage-dependent anion-selective channel protein 1 (**VDAC1**)	P21796	30.9/8.62	144	3	12
**Vesicle trafficking**						
610	septin 11 (**SEPT11**)	Q9NVA2	49.7/6.36	105	6	15

Spot (nr.)	Protein function	Accession Number	MW (KDa)/ p*I*	Mascot score	No. of matched peptides	Sequence coverage (%)
**Cell cycle regulation and aging**						
15	eukaryotic translation initiation factor 3, subunit F (**eIF3f**)	O00303	37.7/5.24	99	5	19
34	eukaryotic translation initiation factor 4A3 (**eIF4A3**)	P38919	47.1/6.3	154	12	24
94	proteasome (prosome, macropain) 26S subunit, non-ATPase, 11 (**PSMD11**)	O00231	47.7/6.08	95	7	20
194	poly (rC) binding protein 1 (**PCBP1**)	Q15365	38.0/6.66	110	6	23
**Translation pathway**						
138	prohibitin (**PHB**)	P35232	29.8/5.57	101	5	21
156	ribosomal protein SA pseudogene 58 (**RPSAP58**)		18.7/9.64	126	2	17
239	polymerase I and transcript release factor (**PTRF**)	Q6N2I2	43.5/5.51	82	5	12
**Energy metabolism**						
91	UDP-glucose 6-dehydrogenase (**UGDH**)	O60701	55.7/6.73	114	7	16
99	citrate synthase, mitochondrial (**CS**)	O75390	51.9/8.45	90	5	10
109	isocitrate dehydrogenase 1 (NADP+), soluble (**IDH1**)	O75874	35.9/6.53	85	5	12
157	fructose-biphosphate aldolase A (**ALDOA**)	P04075	39.8/8.30	163	6	19
166	glyceraldehyde-3-phosphate dehydrogenase (**GAPDH**)	P04406	36.2/5.29	356	6	23
180	aldo-keto reductase family 1, member A1 (aldehyde reductase) (**AKR1A1**)	P14550	36.9/6.32	117	6	18
193	glucose-6-phosphate dehydrogenase (**G6PD**)	P11413	59.8/6.39	121	7	15
250	chain A, structure of human glutamate dehydrogenase-Apo form (**1L1F_A**)	P00367	56.3/6.71	187	12	30
540	ATP synthase, H^+ transporting, mitochondrial F1 complex, beta polypeptide (**ATP5B**)	P06576	56.5/5.26	135	9	27
993	prolyl 4-hydroxylase, alpha polypeptide II (**P4HA2**)	Q05DA4	57.7/5.45	137	8	16
**Lipid metabolism**						
97	enoyl-CoA hydratase, short chain, 1, mitochondrial (**ECHS1**)	P30084	31.8/8.34	114	6	23
139	acetyl-CoA acetyltransferase (**ACAT2**)	Q9BWD1	41.8/6.47	107	3	11
**Muscle development**						
111	lamin A/C (**LMNA**)	P02545	57.7/6.40	133	4	11
126			65.1/6.40	176	12	25
257			74.4/6.57	97	8	14
451			74.3/6.40	107	7	11
**Neuronal differentiation**						
151	dihydropyrimidinase-like 2 (**DPYSL2**)	Q16555	68.6/5.85	84	5	10
483			662.7/5.85	85	6	13
